# Analysis of drug patterns and drug-drug-interactions: associations with physical performance in middle-aged military personnel undergoing rehabilitation– a retrospective cohort study

**DOI:** 10.1186/s40780-025-00422-9

**Published:** 2025-03-03

**Authors:** Jennifer-Daniele Schmitz, Roman Korte, Andreas Lison, Joachim Gerß, Christoph Schulze

**Affiliations:** 1 Department of Orthopaedic Surgery, University Medicine Rostock, Doberaner Str. 152, 18057 Rostock, Germany; 2 The Bundeswehr Center for Sports Medicine, Dr.-Rau-Allee 32, 48232 Warendorf, Germany; 3 Institute of Biostatistics and Clinical Research, University of Münster, Schmeddingstraße 56, 48149 Münster, Germany; 4 Institute for Physical Medicine and Rehabilitation, Paracelsus Medical University, Müllner Hauptstr. 48, Salzburg, A-5020 Austria

**Keywords:** Medical vocational rehabilitation, Physical performance, Long-term-medication, Drug-drug-interactions, Medication management

## Abstract

**Background:**

Comprehensive medication regimens increase the risk of potential drug-drug interactions, adversely affecting health outcomes regardless of age. This risk is particularly pertinent in the context of medical vocational rehabilitation for middle-aged patients, who aim at facilitating rapid reintegration into employment. Identifying and addressing unfavourable drug regimens may substantially contribute to the effectiveness of interdisciplinary therapeutic interventions.

**Methods:**

The retrospective cohort study was conducted among middle-aged soldiers diagnosed with post-traumatic stress disorder and at least one physical impairment and long-term medication intake. Patient records were analysed to investigate the nature of the medication such as the number of drugs and distribution according to the anatomical therapeutic code classification and drug-drug interactions in relation to bicycle ergometry performance.

**Results:**

A substantial majority (73.2%) of all patients enrolled were prescribed an average of 3.0 (± 2.0) long-term medications per person. All patients received treatments containing ATC N drugs, which exert antidepressant properties. On average, each patient encountered the possible risk of 1.7 (± 1.3) drug interactions. Patients administered at least two ATC N drugs exhibited reduced maximum performance compared to controls. Conversely, patients receiving at least two drugs, wherein only one drug classified as ATC N, did not demonstrate significant performance differences from the control group. Notably, treatments incorporating selective monoamine reuptake inhibitors significantly reduced maximum performance relative to controls. The risk for potential drug-drug interactions, particularly those leading to QT interval prolongation, accounted for 47.5% of interactions involving ATC N drugs. Patients exclusively exposed to potential QT-prolonging interactions exhibited significantly reduced maximum performance compared to controls as well as patients who experienced different potential interactions.

**Conclusion:**

Potential drug-drug interactions and disadvantageous drug combinations were prevalent among middle-aged adults with psychiatric disorders and may hinder a positive prognosis for physical fitness. The findings of this study underscore the importance of personalized medication management and continuous monitoring to mitigate negative impacts. Clinicians should diligently review patients’ medication records and adjust therapies accordingly to prevent adverse drug reactions. Proactive strategies, such as regular medication reviews and drug-drug interaction screening tools, may be essential for optimizing therapeutic efficacy and maintaining physical performance.

## Introduction

The primary objective of medical vocational rehabilitation (MVR) is the prompt reintegration into employment [1–3]. Active vocational integration can be facilitated through various modifications and adjustments, such as changes in the workplace environment or workflows, as well as shifts in personal attitudes. This requires a decisive strategic approach within the context of MVR that emphasizes the importance of maintaining work capacity [4, 5]. In addition to personal factors, various medical factors can influence the return to work including co-morbidities, the extent and type of impairment and communication issues between healthcare providers [4]. A review of risk factors pointed out that people were less likely to return to work if they had suffered from intense pain than moderate, long-lasting treatment before the rehabilitation program or depression as a co-morbidity [6, 7]. Particularly for military personnel exposed to deployment-related incidents, these risk factors are more likely to concurrently manifest with significant severity. The management of primary and secondary health conditions and comorbidities associated with complex psychological impairments often require a large number of medications. Given that drug therapy is a crucial component in the treatment of both somatic and mental disease and disabilities, it makes sense to consider it a medical factor that may potentially influence vocational rehabilitation outcome. The identification of drug-related problems (DRPs) or inappropriate medication patterns is a crucial step in medical treatment to deprescribe, eliminate or at least be aware of their existence. For the military profession this might apply to impaired readiness for duty. Adverse effects, adverse drug reactions (ADRs) or other DRPs that occur in other organ systems concurrently carry potential risks for physical and psychological performance [8, 9]. ADRs are characterized as any harmful, undesirable, or unintended effect of a therapeutic substance, whether anticipated or unanticipated, occurring at doses employed for prevention, diagnosis, treatment of diseases, or for altering physiological functions [10]. Drug-drug interactions (DDIs) display an important contributor to ADRs. DDIs can either be of pharmacokinetic or pharmacodynamic nature. While pharmacokinetic interactions involve changes in absorption, distribution or elimination, pharmacodynamic interactions involve synergism and antagonism at the site of action [11, 12]. A recent study revealed that middle-aged rehabilitation patients on complex medication regimens associated with potential DDIs, showed reduced performance on bicycle ergometry compared to controls [13]. However, it is yet to investigate the nature of the medication regimens. To address this, we analyzed both the quality and quantity of the medications to gain insights into prevalent DDIs and medication patterns.

## Methods

### Aim

This study aims to analyze drug patterns and DDIs in middle-aged military personnel undergoing rehabilitation and to assess their impact on physical performance. The findings from this study may raise awareness among healthcare providers about different medications prescribed to middle-aged military personnel undergoing MVR and will also improve their understanding of undesired drug patterns associated with decline in physical performance. Increased awareness may support practitioners to identify inappropriate medications and deprescribe them effectively and safely.

### Study design and participants

This cross-sectional retrospective study enrolled active and former soldiers diagnosed with a primary psychological disorder indicative of posttraumatic stress disorder (PTSD) and complex musculoskeletal system impairments serving the German Armed Forces between 2011 and 2020. Each individual underwent a single bicycle ergometry session during the 3-week rehabilitation period. This assessment was typically conducted on the first, second, or third day of rehabilitation or occasionally took place after the initial interviews, which were part of the physician’s general evaluation to identify physical and mental rehabilitation needs.

Inclusion criteria: (1) Patients demonstrated a significant risk to fitness for duty or ability to perform previous occupational tasks due to physical impairment and PTSD and were therefore eligible to undergo MVR; (2) patients volunteered to participate in this study; (3) possibility to clearly assign each patient to medication group. All patients in the medication group were prescribed long-term medication (LTM) for preventing or alleviating a chronic condition. The term long-term medication, as utilized in this study, refers to medications prescribed for extended durations, with a minimum frequency of intake of at least once daily, depending on the specific indication, drug type, dosage, and pharmacological properties, in accordance with the prescription plan. These medications were prescribed to manage one or more health conditions, including chronic diseases requiring lifelong treatment. Eligibility for inclusion in the study required that the prescribed medication regimen had been initiated at least three months prior to the performance diagnostic assessment. Consequently, all patients enrolled in the study and assigned to the medication group had been provided with at least one prescribed medication at baseline. Therefore, patients were taking the medication on the day of the assessment.

The duration of medication use was measured by having patients bring their medication or prescription plans provided by their doctors to initial doctors’ appointments within the first days of rehabilitation. In cases where patients had recent surgeries or were discharged from the hospital shortly before rehabilitation, they brought their discharge papers. Additionally, patients were interviewed by the rehabilitation physicians regarding their medication, including the drugs used, dosage, frequency, and the time of first administration. This information was documented in a paper file during the initial rehabilitation assessment.

**Fig. 1:**
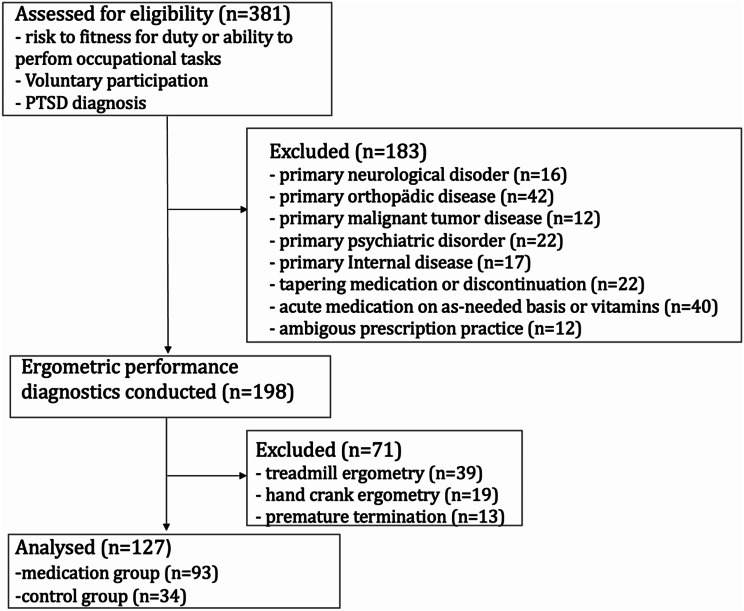
Flow chart of patient selection in this cross-sectional retrospective cohort study between 2011 und 2020

Similarly, patients assigned to the control group without medication did not take any long-term medication for at least three months before the assessment. Moreover, patients must require somatic rehabilitative measures (4); patients must not be diagnosed with cancer or other mental illnesses apart from PTSD (5); patients must attend performance diagnostic measurements (6). Detailed information on the selection process of patients is shown in Fig. 1. A total of 127 patient records were analyzed. Medication data encompassed the name of the active ingredient, dosage and frequency of administration. Drugs were categorized according to their respective anatomical main groups and therapeutic subgroup (ATC classification) to classify and analyze data on drug usage and its effects as well as adverse effects. DDIs were identified using Scholz database (as of 2021/22) and were then categorized as moderate or severe according to the software. Interactions were classified on the basis of the international consensus of potential clinically significant drug interactions in the elderly population [14].

### Cycling ergometry

The maximum performance per kilogram of body weight (p-max. in Watts [W]) and performance at a lactate level of 4 mmol/L in capillary blood (p-4-mmol/l-Lac. [W]) were evaluated using bicycle ergometry “Schiller ERG 911 S plus” (Schiller Medizintechnik, Feldkirchen, Germany). Load adjustments were made according to the guidelines of the German Association for Sports Medicine and Prevention [15]. The test followed a step profile with a 50-Watt increase every three minutes. Maximum performance was defined as the point at which the test was terminated due to subjective stress limits. Capillary blood lactate concentration was measured at three-minute intervals during the test [16]. The “Winlactat” software (Mesics GmbH, Münster, Germany) was used to calculate both the maximum power output and the power level at which the lactate concentration exceeded 4 mmol/L (p-4-mmol/l-Lac.). The detailed procedure for this measurement is documented elsewhere [16].

### Statistical analysis

Statistical analysis was conducted using SPSS Statistics Version 29 (IBM, Armonk, USA) and Scholz database (ePrax GmbH, Munich, Germany). The normal distribution was assessed using Q‒Q plots and Shapiro‒Wilk tests. Descriptive and inferential nonparametric procedures were applied. In descriptive analyses the sample size (N), median (Mdn), and interquartile range (IQR) were calculated by the patients’ medication status during initial rehabilitation. Differences in performance in relation to medication were analyzed using Mann‒Whitney U and Kruskal‒Wallis tests with multiplicity adjustment according to Bonferroni. The association between performance parameters and medication use were investigated using Spearman bivariate correlation analyses. The local significance level was set to α = 5%. Two-sided P values were considered statistically significant in case of *P* ≤ 0.05. GraphPad Prism 6 (GraphPad Software, Inc., California, USA) was used to draw the figures.

## Results

Out of the initial pool of 381 patients, 127 (males: 121 (95.3%)/females: 6 (4.7%)) met the specified inclusion criteria. A total of 93 (73.2%) patients were taking medication, whereas the control group consisted of 34 (26.8%) patients. A comprehensive medication regimen was noted for 42 patients (33.1%). The specific demographic characteristics of this cohort and corresponding comparisons are presented in Table 1.

**Table 1:** Characteristics of patients enrolled (*n* = 127)

Variables	PTSD (*N* = 93)		Control (*N* = 34)		*P*-value
	Mdn	IQR	Mdn	IQR	
Age (years)	38	36–41	37	36–42	0.515
Weight (kg)	96.0	91.4-101.1	93.7	84.8-100.9	0.285
Height (cm)	180.0	180.0-182.0	180.0	179.0-182.0	0.520
BMI (kg/m^2^)	29.3	28.2–30.5	29.6	27.8–31.4	0.417
WHtR	0.56	0.55–0.60	0.54	0.53–0.58	0.546

Data presented as Median (Mdn), Interquartile range (IQR), number (n); p-value: Mann-Whitney U-test

Upon comparing the demographic data across the entire population, no significant differences were detected in any parameter.

### Long-term medication and drug-drug interactions

**Fig. 2:**
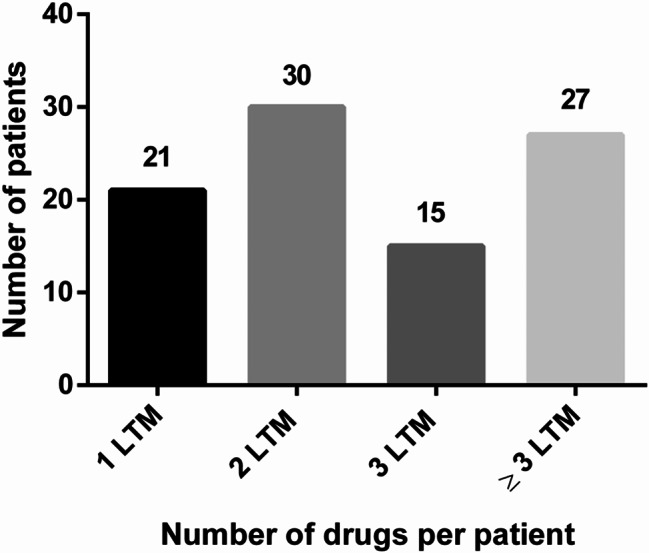
Distribution of the number of long-term medication (LTM) (*N* = 283) across the entire cohort (*N* = 93)

A total number of 283 drugs were identified to be taken on a long-term basis, with an average of 3.0 (± 2.0) drugs per patient. Additionally, 122 potential DDIs were detected. The distribution of drugs per patient is shown in Fig. 2.

All medication groups were compared to a control group, which consisted of 26.8% of all included patients, that did not take any LTM. While primary diagnosed with PTSD, these patients exhibited comorbidities that were predominantly from orthopedic or surgical origin, such as chronic or functional back pain, lumbar and cervical spine syndromes, compartment syndrome, impingement syndrome of the shoulder, and postoperative complaints (52.9%). Additional comorbidities were of internal-orthopedic origin (18.6%) or purely internal origin, such as arterial hypertension, type 2 diabetes mellitus, hyperlipidemia, hyperuricemia, or obesity (14.4%). Moreover, psychiatric-internal complaints were observed, including attention-deficit disorders, recurrent depressive episodes, and concurrent obesity, lipid metabolism disorders, or metabolic syndrome (14.1%).

**Fig. 3:**
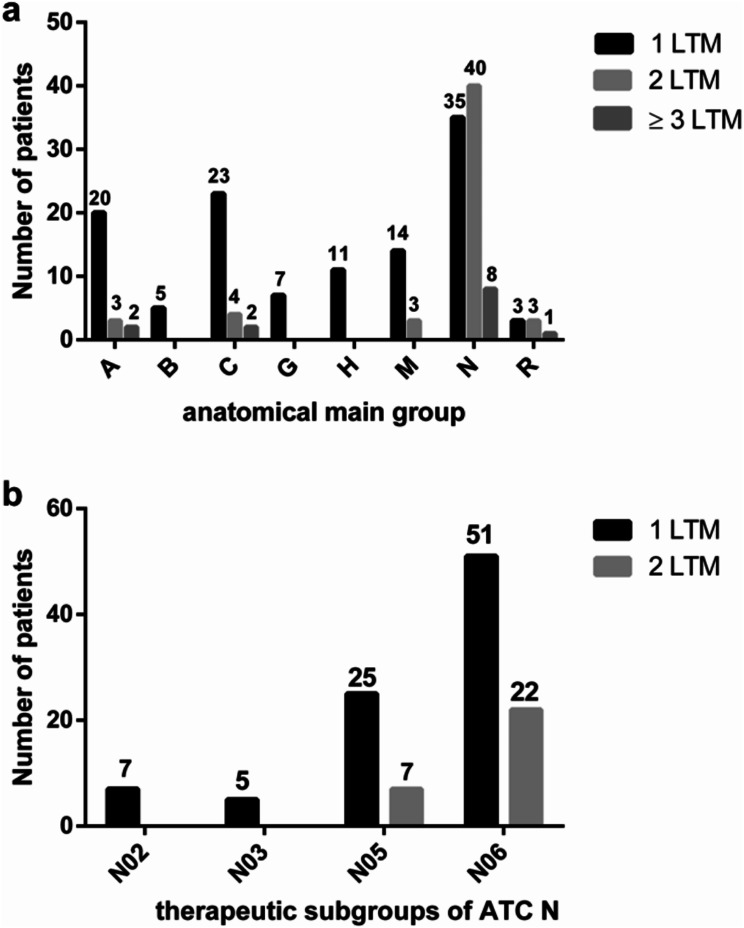
Allocation of all prescribed LTM categorized according to their anatomical main group (**a**) and the therapeutic subgroups for ATC N (**b**). The number of patients whose medication included one, two, three or more drugs within each anatomical main group is depicted in (**a**). The prescribed LTMs were assigned to eight ATC main groups, targeting the following organ systems: A = Alimentary Tract and Metabolism, B = Blood and Blood Forming Organs, C = Cardiovascular System, G = Genitourinary System and Sex Hormones, H = Systemic Hormones (excluding Sex Hormones and Insulin), M = Musculoskeletal System, N = Nervous System, R = Respiratory Tract (**a**). A detailed depiction of the subdivision of ATC N into the therapeutic subgroups N02 (Analgesics), N03 (Antiepileptics), N05 (Antipsychotics), and N06 (Psychoanaleptics) is shown in (**b**) as the number of patients whose medication included one or two drugs within each therapeutic subgroup

To facilitate systematic comparison and analysis of drug use patterns, the drugs were categorized based on their therapeutic and chemical properties according to the Anatomical Therapeutic Chemical (ATC) Classification. The total number of drugs was assigned to eight different main ATC groups as well as four therapeutic subgroups, as presented in Fig. 3.

Among all patients, 72 (77.4%) patients received medication consisting of at least two long-term drugs, while the medication of 66 (71.0%) patients included drugs assigned to ATC N. The last resulted into 113 potential DDIs. Out of these interactions, 76 (65.0%) were classified as potentially moderate and 41 (35.0%) as potentially severe. 16 patients (24.2%) were at intermediate risk of experiencing an interaction due to their medication, 18 patients (27.3%) of the drug combinations posed low risk, 13 (19.7%) patients were identified with both DDI from intermediate and low risk, while 19 (28.8%) patients did not experience any DDI. On average, each patient experienced 1.7 (± 1.3) drug interactions. Figure 3 illustrates the potential effects of DDIs and their frequency of occurrence observed.

### Performance in relation to LTM

#### ATC N – drugs that target the nervous system

Data analysis revealed that 80 out 93 patients (86.0%) took at least one drug that was systematically assigned to ATC N, which target the central nervous system. Comparing all main ATC groups identified in this cohort with each other, drugs assigned to ATC N made up the largest share in terms quantity. The group of patients taking ATC N medications included 63.0% of all enrolled patients, who consumed an average of 3.1 ± 2.1 long-term medications (LTM). Overall, 17.6% of the patients took one LTM, 36.4% took two LTMs, and 44.0% took three or more LTMs. Comorbidities were primarily of a purely orthopedic or surgical nature (43.8%), orthopedic-internal origin (26.3%), or orthopedic-neurological origin (3.8%), such as neck pain and back pain occurring alongside migraines. Additional comorbidities included psychiatric-orthopedic conditions (5.0%) and psychiatric-internal conditions (21.1%).

For the therapeutic subgroups it is N06 which made up the largest share and is the subgroup that hosts the drugs explicitly approved for PTSD treatment in Germany. In total, 22 patients (27.5%) received one drug approved for PTSD in Germany and 50 patients (62.5%) received a selective monoamine reuptake inhibitor, which was either a selective serotonin reuptake inhibitor (SSRI), a serotonin norepinephrine reuptake inhibitor (SNRI) or a selective serotonin norepinephrine reuptake inhibitor (SSNRI), which exhibit antidepressive properties.

Overall, patients who consumed three or more drugs obtained significantly poorer p-max. than control (*N* = 42, 217 (200–250) W vs. *N* = 34, 250 (225–267) W, *P* = 0.021). Maximum performance regime was inversely correlated with inclining numbers of drugs belonging to ATC N in each medication regime leading to a complex medication containing two or more ATC N drugs among others (Table 2). Moreover, it was found that patients receiving medication that contained two or more drugs, whereby at least two drugs classified as ATC N exhibited a significantly poorer maximum performance than control (*N* = 39, 208 (221–255) W vs. *N* = 34, 250 (225–267) W, *P* = 0.024), while patients taking an equivalent number of drugs with only one drug classified as ATC N did not differ from controls. P-4-mmol Lac. was not significantly different in any of these cases. The number of drugs belonging to ATC N in each medication regime were inversely correlated with maximum performance, albeit weakly (Table 2).

**Table 2:** Spearman correlation of p-max. And p-4-mmol Lac. With ATC main group N, presence of serotonin monoamine reuptake inhibitors And potential occurrence of QT interval prolongation

		ATC *N*	SMRI	DDI QT
p-max.	Spearman rho	− 0.215^*^	− 0.312^**^	− 0.319^**^
	Sig. 2-tailed	0.022	< 0.001	0.003
	N	114	114	86
p-4-mmol Lac.	Spearman rho	− 0.120	− 0.257^**^	− 0.229^*^
	Sig. 2-tailed	0.217	0.007	0.041
	N	107	114	107

With ATC N = Drugs belonging to anatomical therapeutic classification N; SMRI = Drugs categorized as selective monoamine reuptake inhibitors; DDI QT = Drug-drug interaction classified as QT interval prolongation. ^*^Correlation is significant at 0.05 level (two-tailed); ^**^correlation is significant at 0.01 level (two-tailed)

### Drugs provoking QTc- interval prolongation

Of all patients with medication, 8 (10%) took drugs that did not include drugs exhibiting QT interval prolongation as a potential adverse side effect, while 36 (44.4%) took one, 26 (32.5%) took two and 10 (12.5%) took three medications known to prolong QT interval. A total of 15.0% of all included patients were assigned to the group of patients experiencing at least one DDI resulting from their overall medication that has potential to prolong QT interval. Patients in this group took an average of 3.3 ± 1.8 medications per person, with 52.6% taking at least two medications and 47.4% taking three or more medications. Patients in this subgroup predominantly suffered from orthopedic and surgical comorbidities (52.6%), including functional musculoskeletal complaints, lumbar spine syndrome, and postoperative complaints following fractures. Additionally, orthopedic-psychiatric complaints (18.4%) were observed, such as lower back pain along with depressive episodes or trauma-related disorders sleep disturbances. Further comorbidities included orthopedic-internal medical conditions (29.0%), which for example involved musculoskeletal pain alongside arterial hypertension, hypo- and hyperthyroidism, hyperlipidemia, and obesity.

**Fig. 4:**
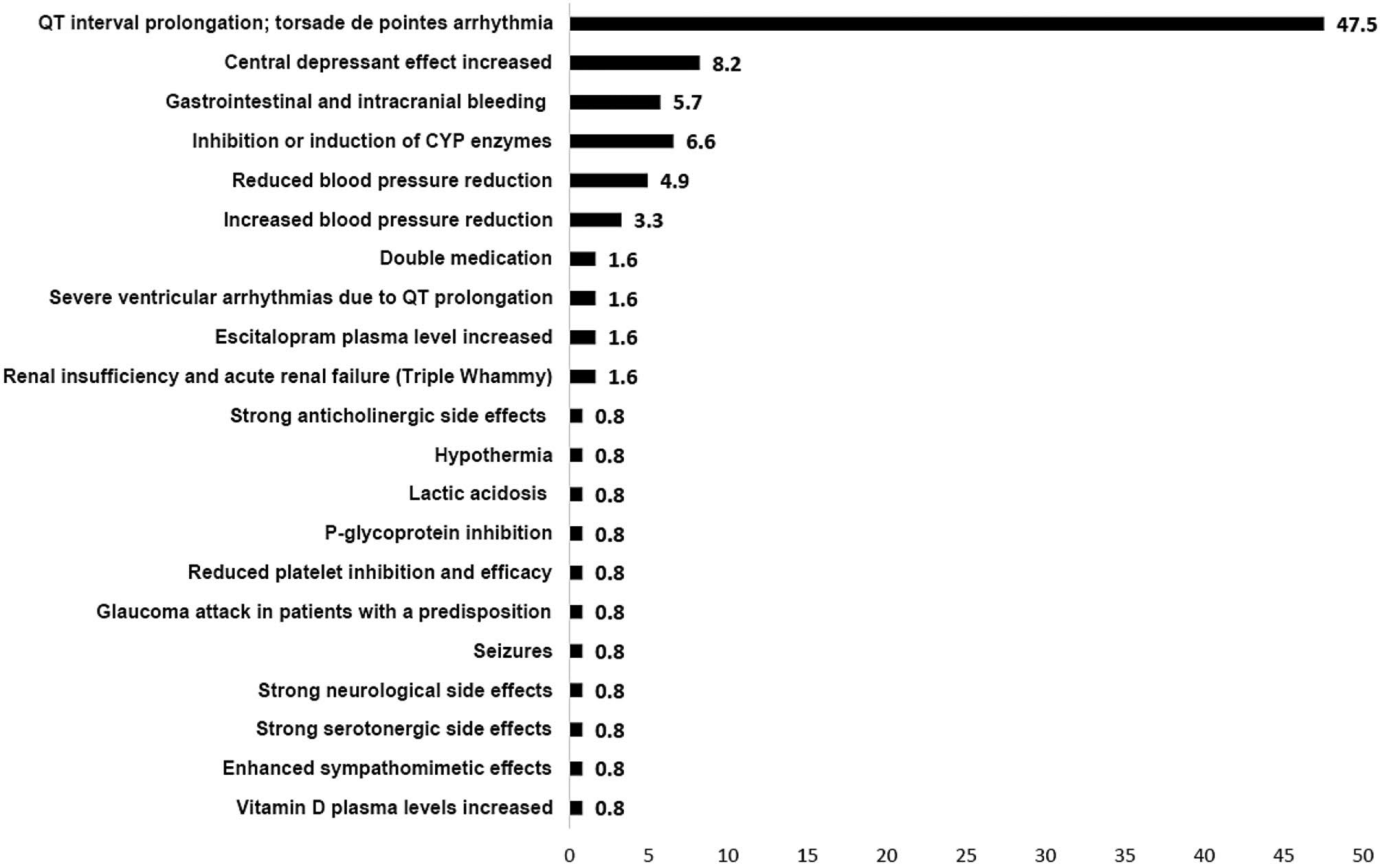
Emerging effects due to potential DDI and the frequency of their occurrence. Data are presented as percentage (%)

As indicated in Figs. 4 and 47.5% of all potential DDIs involving at least one ATC N drug were identified as potential cause of QT interval prolongation. Statistical analysis revealed that patients exclusively exposed to at least one DDI that potentially prolongs the QT interval exhibited significantly reduced maximum performance compared to control (*N* = 19, 208 (175–258) W vs. vs. *N* = 34, 250 (225–267) W *p* = 0.021). The maximum performance correlated negatively with the occurrence of QT interval prolonging drugs in a complex medication regime (Table 2). Drugs that have been determined to have interactions that increase the risk of developing QT interval prolongation was included in the appendix ( Table 3).

**Table 3:** List of drugs that have been determined to have interactions that increase the risk of developing QT interval prolongation

DDI	Degree of serverity	Effect of DDI
Trimipramine Venlafaxine	severe	QT interval prolonged, risk of Torsades de pointes
Sertraline Trimipramine	severe	Trimipramine metabolism slowed; risk of QT interval prolongation, Torsades de pointes
Promethazine Rizatriptan	intermediate	QT interval prolonged, risk of Torsades de pointes
Amitriptyline Lithium	severe	QT interval prolonged, risk of Torsades de pointes
Melperone Lithium	severe	QT interval prolonged, risk of Torsades de pointes
Amitriptylin Bupropion	severe	Amitriptyline metabolism slowed; risk of QT interval prolongation, Torsades de pointes
Amitriptyline Melperone	severe	QT interval prolonged, risk of Torsades de pointes
Chlorprothixene Pregabalin	intermediate	QT interval prolonged, risk of Torsades de pointes
Pregabalin Pantoprazole	intermediate	QT interval prolonged, risk of Torsades de pointes
Chlorprothixene Pantoprazol	severe	QT interval prolonged, risk of Torsades de pointes
Escitalopram Hydrochlorothiazide	severe	Arrhythmia risk of QT interval prolongation increased in hypokalemia
Sertraline Quetiapine	intermediate	QT interval prolonged, risk of Torsades de pointes
Aripiprazole Hydrochlorothiazide	intermediate	Arrhythmia risk of QT interval prolongation increased in hypokalemia
Sertraline Hydrochlorothiazide	intermediate	Arrhythmia risk of QT interval prolongation increased in hypokalemia
Aripiprazole Sertraline	severe	Aripiprazole plasma levels increased due to inhibition at CYP2D6, QT interval prolongation
Amlodipine Aripiprazole	intermediate	Aripiprazole plasma levels increased due to inhibition at CYP3A4
Trimipramine Escitalopram	severe	QT interval prolonged, risk of Torsades de pointes
Escitalopram Quetiapine	severe	Risk of severe ventricular arrhythmias due to QT prolongation
Omeprazole Quetiapine	intermediate	QT interval prolonged, risk of Torsades de pointes
Mirtazapine Quetiapine	intermediate	QT interval prolonged, risk of Torsades de pointes
Formoterol Pregabalin	intermediate	QT interval prolonged, risk of Torsades de pointes
Venlafaxine Pregabalin	intermediate	QT interval prolonged, risk of Torsades de pointes
Venlafaxine Formoterol	intermediate	QT interval prolonged, risk of Torsades de pointes
Formoterol Esomeprazole	intermediate	QT interval prolonged, risk of Torsades de pointes
Venlafaxine Promethazine	severe	QT interval prolonged, risk of Torsades de pointes
Mirtazapine Promethazine	intermediate	QT interval prolonged, risk of Torsades de pointes
Sertraline Trazodone	intermediate	QT interval prolonged, risk of Torsades de pointes
Fluoxetine Chlortalidone	intermediate	Arrhythmia risk of QT interval prolongation increased in hypokalemia
Quetiapine Fluoxetine	severe	QT interval prolonged, risk of Torsades de pointes
Omeprazole Quetiapine	intermediate	QT interval prolonged, risk of Torsades de pointes
Loperamide Omeprazole	intermediate	QT interval prolonged, risk of Torsades de pointes
Loperamide Escitalopram	severe	QT interval prolonged, risk of Torsades de pointes
Bupropion Trimipramine	severe	Trimipramine metabolism slowed; risk of QT interval prolongation, Torsades de pointes
Quetiapine Pantoprazole	intermediate	QT interval prolonged, risk of Torsades de pointes
Venlafaxine Pantoprazole	intermediate	QT interval prolonged, risk of Torsades de pointes
Venlafaxine Indapamide	intermediate	Arrhythmia risk of QT interval prolongation increased in hypokalemia
Venlafaxine Mirtazapine	severe	QT interval prolonged
Mirtazapine Indapamide	intermediate	Arrhythmia risk of QT interval prolongation increased in hypokalemia
Pantoprazole Indapamide	intermediate	Arrhythmia risk of QT interval prolongation increased in hypokalemia
Venlafaxine Maprotiline	intermediate	Maprotiline metabolism inhibited by cytochrome P450 CYP2D6, plasma levels increased
Pantoprazole Mirtazapine	intermediate	QT interval prolonged, risk of Torsades de pointes
Escitalopram Mirtazapine	severe	QT interval prolonged, risk of Torsades de pointes
Escitalopram Pantoprazole	severe	QT interval prolonged, risk of Torsades de pointes
Mirtazapine Formoterol	intermediate	QT interval prolonged, risk of Torsades de pointes
Mirtazapine Trimipramine	intermediate	QT interval prolonged, risk of Torsades de pointes
Trimipramine Formoterol	intermediate	QT interval prolonged, risk of Torsades de pointes
Pantoprazole Promethazine	intermediate	QT interval prolonged, risk of Torsades de pointes
Omeprazole Trazodone	intermediate	QT interval prolonged, risk of Torsades de pointes
Omeprazole Venlafaxine	intermediate	QT interval prolonged, risk of Torsades de pointes
Venlafaxine Trazodone	severe	QT-Intervall prolonged
Venlafaxine Quetiapine	severe	QT interval prolonged, risk of Torsades de pointes
Venlafaxine Solifenacin	severe	QT interval prolonged, risk of Torsades de pointes
Pantoprazole Solifenacin	intermediate	QT interval prolonged, risk of Torsades de pointes
Quetiapine Solifenacin	severe	QT interval prolonged, risk of Torsades de pointes
Quetiapine Pantoprazole	intermediate	QT interval prolonged, risk of Torsades de pointes

Patients who experienced different DDI at the same time did not exhibit a decline in performance compared to the control group. There were no significant differences in p-4mmol Lac. levels in any of the groups in terms of maximum performance on bicycle ergometry, however it correlated negatively with the occurrence of potential QT interval prolonging drugs in a complex medication regime (Table 2), although we did not observe any abnormal cardiac changes by ECG.

### Selective monoamine reuptake inhibitors

A total of 35.5% of all included patients were assigned to the SMRI group. These patients took an average of 3.1 ± 2.0 medications per person, with 45.5% taking two medications and 54.5% taking three or more medications. Patients in this subgroup predominantly suffered from orthopedic and surgical comorbidities (32.7%), such as functional back pain, various forms of osteoarthritis, knee joint pain, postoperative complaints following upper or lower leg fractures, or polytrauma. Additionally, orthopedic complaints were present along with internal medical conditions, such as hyperlipidemia, arterial hypertension, or obesity (28.8%). Further comorbidities included purely internal medical conditions (15.4%) as well as psychiatric and orthopedic complaints (23.1%), such as depressive episodes with low back pain or panic disorders with functional back pain.

In cases where the patient’s medication contained at least two drugs with one being SSRI, SNRI or SSNRI, the patients achieved significantly lower maximum performance compared to controls (p-max. *N* = 44, 208 (200–250) W vs. *N* = 34, 250 (225–267) W, *P* = 0.004) with a trend towards significance for p-4-mmol Lac. (*N* = 44, 158 (129–174) W vs. *N* = 34, 171 (148–191) W, *P* = 0.052). For all other cases characterized by the absence of a selective monoamine reuptake inhibitor (SMRI) but the presence of one other ATC N category drug, no significant differences compared to control were identified. Both maximum performance and performance at 4 mmol/l lactate threshold in capillary blood correlated negatively with the presence of selective monoamine reuptake inhibitors in the course of no medication, medication lacking selective monoamine reuptake inhibitors, and medication including SMRI.

## Discussion

### Statement of key findings

This study aimed to analyze and evaluate medication patterns and DDIs among middle-aged military personnel undergoing rehabilitation, specifically in relation to their physical performance. By examining the quality and quantity of the medications used, we sought to gain a deeper understanding of how these factors may influence performance outcomes, particularly in the context of complex medication regimens. The majority of patients were on multiple medications, which highlights the prevalence of polypharmacy, which denotes concurrent long-term use of three or more drugs within this cohort [17].

Of all patients with medication intake, we observed 86.0% to be prescribed with at least one drug categorized as ATC N. Among all ATC groups, ATC N drugs were most frequently prescribed, with subgroup N06, that encompasses antidepressants, representing the largest proportion. Specifically, 27.5% of patients diagnosed with PTSD received drugs approved for PTSD treatment in Germany, while 62.5% received selective monoamine reuptake inhibitors (SSRI, SNRI, SSNRI), which are not exclusively approved for PTSD but are indicated for forms of depressive disorder. Moreover, we observed a significant decline in maximum performance among patients taking at least two drugs from ATC N as well as in cases where medication included SSRI, SNRI, or SSNRI compared to controls. There was an inverse relationship between the number of ATC N drugs and maximum performance.

PTSD frequently co-occurs with comorbid diagnoses, particularly depressive disorders, which may require additional pharmacotherapeutic support [7, 18, 19]. Due to the overlap and the fact that only three medications are currently approved for the treatment of PTSD, to which patients may respond differently based on intra-individual characteristics or suffer from pronounced side effects, antidepressants and/or antipsychotics represent potential treatment options that prescribing physicians, in agreement with the patients, may consider [20].

Regarding drug interactions, 47.5% of potential interactions involving ATC N drugs were identified as capable of prolonging the QT interval. Patients exclusively exposed to QT-prolonging DDIs exhibited significantly reduced maximal performance compared to controls. Maximal performance showed a negative correlation with the presence of QT-prolonging drugs in comprehensive medication regimens.

These findings suggest potential associations between certain drugs classified as ATC N drugs, particularly SSRIs and/or SNRIs and physical performance metrics. They highlight potential implications on patient outcomes, particularly concerning physical performance in the context of possible QT interval prolonging DDI in middle-aged rehabilitation patients.

Chemically, antidepressants that act as SSRI and SNRI (e.g. citalopram/escitalopram) among others can block HERG channels, inhibiting rapid potassium influx and prolonging the repolarization phase, thereby increasing the risk for possible QT-interval prolongation [21, 22]. In general, the effects of antidepressants and antipsychotics to the ATC N category on physical performance are associated with their lack of receptor specificity, rendering them “dirty drugs” [23]. While a broad receptor activity may enhance therapeutic effects, it also contributes to a wide range of side effects. For example, TCAs and typical antipsychotics target multiple receptors, including serotonin, dopamine, and histamine receptors [23]. Their action on central nervous system receptors can lead to fatigue and muscle weakness [23]. Extrapyramidal side effects, such as tremor and muscle stiffness, arise from D2 dopamine receptor blockade [24]. Furthermore, cardiovascular fitness may be compromised by orthostatic hypotension, a well-documented, common side effect as well as metabolic changes related to weight modifications, which can result in fatigue and/or anhedonia [25]. Most of these effects can be exacerbated by pharmacokinetic or pharmacodynamic interactions and are particularly sensitive to electrolyte imbalances [11, 12].

## Interpretation

### Performance in relation to LTM

Recent literature has highlighted a significant decrease in physical fitness among depressive patients compared to healthy controls, independent of BMI, age, sex, and physical activity [26]. This study extends these findings by identifying a pattern among the patients with LTM. Specifically, these patients exhibited significantly lower maximum performance levels and reduced blood lactate concentrations compared to the controls. The data extracted and analysed from our cohort suggest that factors such as age, weight, height, waist-to-height ratio (WHtR), and BMI did not significantly contribute to the observed decline in maximum performance. These results are consistent with recently published literature [26], indicating that neither PTSD presence nor the examined anthropometric characteristics are primary determinants of reduced maximum performance. The categorization of medications according to the ATC classification provides a systematic approach to analyzing drug use patterns. The high prevalence of drugs in ATC group N indicates a significant focus on medications affecting the nervous system. The results indicate no significant difference in performance metrics among patients whose medication includes two or more drugs, with only one drug classified as ATC N, wherein two or more ATC N drugs did. This suggests that drugs targeting the same organ system may be associated with reduced performance when administered simultaneously. This reduction in performance could be attributed to the reinforcement of central depressant effects, such as sedation and drowsiness [27], or to pharmacokinetic interactions that alter the metabolism of the drugs [12, 28]. Certain SMRIs exhibit variable bioavailability due to their first-pass metabolism. These drugs are metabolized in the liver via cytochrome P450 enzymes and can either inhibit or induce specific CYP enzymes, leading to increased or decreased blood levels of the involved drugs [27]. TCAs show very high protein binding and have a large volume of distribution due to their lipophilicity [29]. From a pharmacodynamic perspective, the interaction of these drugs can be unpredictable [11]. Antidepressants primarily increase the levels of neurotransmitters such as serotonin, norepinephrine, and dopamine in the brain. SSRIs inhibit the reuptake of serotonin, enhancing its synaptic availability [29, 30]. Common side effects include gastrointestinal disturbances, sexual dysfunction, weight gain, and cardiovascular issues like arrhythmias in the case of TCAs [29, 31]. These effects can potentially lead to adverse outcomes such as hypotension or severe central nervous system depression, contrary to the therapeutic objectives [11, 12, 27, 32, 33].

### Drugs provoking QT interval prolongation

The study reveals a considerable risk of moderate and severe DDIs among the patients, whereby QT interval prolongations appeared to be most common DDI of profound severity. QT interval prolongations are known for a wide range of drugs, including many antidepressive and antipsychotic medications [34, 35]. In diagnostics, the heart rate-corrected QT interval is calculated and referred to as the QTc interval. Torsades de Pointes (TdP) is associated with QTc prolongation, which is the heart rate adjusted lengthening of the QT interval [34, 35]. In monotherapy, these cardiac alterations are usually clinically irrelevant, but in combination with other drugs, they can become risky [35, 36]. After evaluating the ECGs recorded during the three-week rehabilitation program for all patients, no abnormal cardiac changes were observed, regardless of medication intake and number of drugs taken. Since this cohort is middle-aged and generally not at risk to suffer from torsade-de-pointes arrythmia, which is associated with an age ≥ 65 years, female gender, substantially low electrolyte levels, heart disease and diuretic use, the results align with the literature [37–39]. However, it was interesting to note that patients who were exclusively exposed to one or more DDIs that developed potential QT interval prolonging effects, achieved significantly reduced maximum performance compared to control. At the same time, patients experiencing a combination of various DDIs did not exhibit a decline in performance compared to the control group. Underlying baseline characteristics such health conditions, age, or the number of medications did not differ between the QT and non-QT group.

This finding suggests that the reduction in performance might be linked to QT prolonging drugs and/or the interaction. Since QT-prolonging drugs represent a large and heterogeneous group of pharmaceutical substances, it is conceivable that these medications possess properties that induce more pronounced performance-impairing side effects such as fatigue, dizziness, tiredness, or vertigo [32]. Moreover, it is possible that the DDIs differ in pharmacodynamic and pharmacokinetic profiles, where some drugs might have additional sedative or cognitive impairing effects. These symptoms can lead to reduced performance without directly impacting cardiopulmonary health. The lack of performance decline in patients experiencing various DDIs suggests that the presence of multiple potential interactions might lead to compensatory mechanisms or balancing effects that mitigate the negative impact on performance [40]. This could be due to the presence of drugs that counteract the adverse effects of QT prolongation or even mimic or neutralize the QT prolongation itself. Another assumption include synergistic or antagonistic effects that modulate the central nervous system in ways that preserve or enhance performance and therefore stabilize overall performance [11].

### Selective monoamine reuptake inhibitors (SMRIs)

SMRIs which inhibit the reuptake of monoamine neurotransmitters are widely used in the treatment of various psychiatric disorders. In Germany, the two SMRIs, Paroxetine and Sertraline, are officially approved for the treatment of PTSD [20, 41, 42]. In this study, 62.5% of patiens in the medication-group were prescribed at least one SMRI, with 27.5% specifically taking Paroxetine or Sertraline. Among these patients whose medication consisted of at least two medications and contained at least one SMRI we observed a significantly lower maximum performance compared to the control group. This decline in performance was not observed in patients with ≥ 2 LTM taking one other ATC N drug that is not SMRI.

Research on the influence of SMRIs on physical activity suggests that alleviation of depressive symptoms might encourage increased physical activity [43]. Comparative studies have shown that different types of SMRIs influence physical activity differently, with patients on SNRIs and Serotonin-dopamine reuptake inhibitor (SDRI) experiencing more pronounced improvements in physical activity compared to those on SSRIs [44, 43, 30]. Given that this study cohort consisted of patients diagnosed with PTSD who took more than one drug or were on a comprehensive medication, we did not expect our results to align with existing literature. Nevertheless, the findings are intriguing, as SMRIs are intended to reduce anxiety and fatigue while enhancing mood and motivation. Clinical considerations for prescription include tailored choices of SMRIs based on the patient’s symptoms, medical history and potential drug interactions. Therefore, it is conceivable that the SMRI chosen might not ideally match the patient’s symptoms or align with previous treatment decisions. Optimal efficacy must to be balanced with tolerability [44, 31]. The difference in efficacy might as well affect the underlying cohort. Due to varying modes of action and inter- and intra-individual pharmacokinetic variability, there is substantial variation in the frequency of adverse drug effects between classes of antidepressants [29,31, 44]. Thus, monitoring for efficacy and side effects as well as careful supervision in cases of combination use, is essential for determine efficacy.

This suggests that comprehensive medication regimen comprising two or more drugs, wherein one belongs to the class of SMRIs, may constitute an adverse drug combination for middle-aged soldiers undergoing MVR.

### Strengths and limitations

To the best of our knowledge, this is the first retrospective cohort-study to identify drug patterns and DDIs present in middle-aged patients undergoing MVR. However, we want to address that this study has limitations. Firstly, due to the retrospective data extraction there was no direct patient contact at any time during data collection. Therefore, the determination of the DDIs were made based on the available records, which did not include timing of administration and dosage. Secondly, there is a substantial gender imbalance among the patients who met the inclusion criteria, with a predominance of male patients. Thirdly, self-procured over-the-counter medication, that was not reported, was not taken into consideration but might add complexity to the overall medication. It might as well be considered that disadvantageous physical health conditions and injuries, either alone or in combination with psychological changes, such as a loss of motivation or social and environmental factors, such as financial stress, lack of support, or difficult living conditions, may influence a patient’s ability or willingness to engage in physical activity can also lead to decreased physical activity [19, 45].

However, the absence of significant differences in anthropometric data between patients with medication and the control group suggests a homogenous distribution within the cohort, indicating that these variables are unlikely to introduce substantial bias into the results.

## Conclusion

This study underscores the role of medication in MVR of middle-aged patients. Specifically, it emphasizes the need for careful evaluation of medication regimens that include more than one drug from ATC N or one SMRI due to their potential to develop pharmacodynamic and/or pharmacokinetic interactions as well as carry out adverse side effects. This evaluation is crucial even if the patient’s overall medication regimen consists of only two drugs. Our observations indicate that polypharmacy may act as a precursor to impaired physical performance, but it is not a prerequisite; two drugs with adverse interactive effects can be sufficient to negatively impact physical performance, even if there is no direct point of reference such as an irregular ECG. In this context, DDIs classified as QT prolongation, that occur exclusively, may potentially serve as a predictor for reduced performance development. Continuous monitoring of drug efficacy and side effects, particularly for SMRIs and QT-prolonging medications, is essential to optimize therapeutic outcomes and maintain physical performance. For implementation this could be proactively addressed via regular medication reviews and use DDI screening tools as well as drug monitoring to identify and manage potential interactions. Moreover, the development and promotion of guidelines for managing multiple drugs including polypharmacy, with a focus on middle-aged physically and mentally impaired patients, could pave the way for optimization in MVR. Another promising approach might be the education of healthcare providers about the risks and the importance of medication reconciliation during every patient visit [46, 47].

## Future research

Further research is needed to understand i.e. the gender disparity observed and address any underlying biases or health access issues. Moreover, for patients using drug combinations expected to prolong the QT interval, further research could focus on confirming specific combinations that are supposed to increase the risk of QT-interval prolongation, even without detectable abnormal cardiac changes on ECG, to assess their potential as predictors of reduced performance development.

## Data Availability

Data is provided within the manuscript. Moreover, datasets used and/or analyzed during the current study are available from the corresponding author on reasonable request, since we have to protect study participant privacy.
